# The perils of planning strategies to increase vitamin C content in plants: Beyond the hype

**DOI:** 10.3389/fpls.2022.1096549

**Published:** 2022-12-19

**Authors:** Mattia Terzaghi, Mario C. De Tullio

**Affiliations:** ^1^ Department of Biosciences, Biotechnologies and Environment, University of Bari "Aldo Moro", Bari, Italy; ^2^ Department of Earth and Geoenvironmental Sciences, University of Bari "Aldo Moro", Bari, Italy

**Keywords:** vitamin C, ascorbate biosynthesis, Smirnoff-Wheeler pathway, dehydroascorbate reductase, ascorbate oxidase

## Abstract

Ever since the identification of vitamin C (ascorbic acid, AsA) as an essential molecule that humans cannot synthesize on their own, finding adequate dietary sources of AsA became a priority in nutrition research. Plants are the main producers of AsA for humans and other non-synthesizing animals. It was immediately clear that some plant species have more AsA than others. Further studies evidenced that AsA content varies in different plant organs, in different developmental stages/environmental conditions and even within different cell compartments. With the progressive discovery of the genes of the main (Smirnoff-Wheeler) and alternative pathways coding for the enzymes involved in AsA biosynthesis in plants, the simple overexpression of those genes appeared a suitable strategy for boosting AsA content in any plant species or organ. Unfortunately, overexpression experiments mostly resulted in limited, if any, AsA increase, apparently due to a tight regulation of the biosynthetic machinery. Attempts to identify regulatory steps in the pathways that could be manipulated to obtain unlimited AsA production were also less successful than expected, confirming the difficulties in “unleashing” AsA synthesis. A different approach to increase AsA content has been the overexpression of genes coding for enzymes catalyzing the recycling of the oxidized forms of vitamin C, namely monodehydroascorbate and dehydroascorbate reductases. Such approach proved mostly effective in making the overexpressors apparently more resistant to some forms of environmental stress, but once more did not solve the issue of producing massive AsA amounts for human diet. However, it should also be considered that a hypothetical unlimited increase in AsA content is likely to interfere with plant development, which is in many ways regulated by AsA availability itself. The present review article aims at summarizing the many attempts made so far to improve AsA production/content in plants, evidencing the most promising ones, and at providing information about the possible unexpected consequences of a pure biotechnological approach not keeping into account the peculiar features of the AsA system in plants.

## 1 Introduction: Is vitamin C the new panacea?

Since its identification as the long sought anti-scurvy factor vitamin C (King and Waugh, 1932; Svirbely and Szent-Gyorgyi, 1932) ascorbic acid (formerly known as hexuronic acid) has been the subject of extensive research. The name ascorbic acid (AsA) literally means “against scurvy”, and early research was mainly oriented to understanding how the deadly disease known as scurvy could be prevented and cured by the newly found molecule, but the biochemical mechanism underlying the beneficial effect of AsA in scurvy prevention was eventually discovered only much later (Stone and Meister, 1962). Scurvy is a complex disease involving several concomitant malfunctions, apparently all caused by the inactivation of different enzymes belonging to the large family of 2-oxoglutarate-dependent dioxygenases (De Tullio, 2012). With the general improvement of life conditions, scurvy became a rare disease for a large part of the world population, although some scattered cases still occur (Amisha et al., 2022). Nowadays, vitamin C is very popular, but definitely not because of its original role as the anti-scurvy factor. The reasons for such popularity trace back to the ‘70s and ‘80s of the last century, when the double-Nobel laureate (for Chemistry in1954, and for Peace in 1962) Linus Pauling established collaborations with the biochemist Irwin Stone and the clinician Ewan Cameron. On the basis of limited clinical data and his own experience, Pauling claimed that “megadoses” (up to 18 grams per day)- of vitamin C are effective against many different pathologies: from the common cold to cancer (Pauling, 1986). Pauling’s megadoses largely exceed the daily amount of vitamin C currently recommended by official international bodies and health organizations. For example, the European Food Safety Authority recommends a daily intake in the milligram range, differentiated according to age and specific requirements (**Table 1**). In parallel with Pauling’s promise of a long and healthy life with vitamin C, the Free Radical Theory of Ageing received increasing consideration. Initially proposed in the 1950s by the gerontologist Denham Harman (Harman, 1956), the theory, based on the concept that ageing results from oxidative stress, became more and more popular as it provided an apparently simple molecular explanation to the complexity of the ageing process. Later studies further suggested that free radicals (but also hydrogen peroxide, which is not a free radical) are involved in the pathogenesis of an array of diseases, stressing the importance of antioxidants as a tool to counteract the damage caused by reactive oxygen species (Dormandy, 1978). Over the years and the decades, the popularization of the Free Radical Theory of Ageing and Disease has led to a dramatic oversimplification, inducing in the general public the belief that antioxidants, and in particular vitamin C, can protect us from almost any harm. Unfortunately, this is not true. More and more studies contradict the initial assumptions of the Theory (Gladyshev, 2014). We now know that generically increasing “antioxidant defenses” can even have detrimental effects, as reactive oxygen species also have essential roles as signaling molecules (Mittler, 2017). Thus, massive removal of reactive oxygen species by antioxidants is not only impossible, but also not desirable. In spite of this simple consideration, the market of antioxidant supplements is worth several billion dollars, and a boost in sales occurred during the recent Covid-19 pandemic (Evans, 2020). Vitamin C requirement by consumers is likely to further increase in the next years.

**Table 1:** Ascorbic acid requirement (mg/day) in different subsets of the population, according to the European Food Safety Authority. http://multimedia.efsa.europa.eu/drvs/index.html.

	Average Requirement* (mg)	Population Reference Intake** (mg)
Adults (≥18 years)	80	95
Infants 7-11 months	–	20
Children (1-3 years)	15	20
Children (7-10 years)	40	45
Adolescents (15-17 years)	75	90
Pregnant women (≥18 years)	–	105
Lactating women (≥18 years)	140	155

*The average requirement (AR) refers to the intake of a nutrient that meets the daily needs of half the people in a typical healthy population.**The population reference intake (PRI) is the intake of a nutrient that is likely to meet the needs of almost all healthy people in a population.

## 2 Why increasing vitamin C content in plants

An effective and inexpensive industrial method for vitamin C production was developed as early as 1933 by Reichstein, and additional synthetic methods have been proposed thereafter (Pappenberger and Hohmann, 2014). Nevertheless, plants remain the main source of vitamin C for human consumption. It is worth mentioning that most animal species do not need AsA supplementation, as they can produce it themselves using a well-known biosynthetic pathway (Duque et al., 2022). Humans and some other primates, bats, some birds, and a few more species have lost this capability due to the loss of function of the gene encoding l-gulono-1,4-lactone oxidase, the enzyme catalyzing the final step in AsA biosynthesis (Nishikimi and Yagi, 1991).

Although no difference in reactivity and effectiveness can be observed between “natural” and “man-made” AsA, time and efforts were necessary to make vitamin C produced with the Reichstein method appealing to consumers (Bächi, 2008). Still today, it is generally assumed that “natural” vitamin C is always preferable, possibly because in plant extracts other beneficial factors (*e.g.* bioflavonoids) are also present. Unfortunately, this opinion is not substantiated by experimental data, as there is no evidence of differences in the bioavailability of synthetic *versus* food-derived vitamin C in humans (Carr and Vissers, 2013). Anyway, the popularity of vitamin C is now so high, that producing plants able to provide us with AsA megadoses in a few bites seems a desirable goal: the more the better. As discussed in the next sections of this article, this is not an easy goal to reach, and many attempts failed. On the other hand, in some cases plants possessing even slightly higher AsA content proved more resistant to abiotic stress conditions as compared to controls. Such findings opened a different opportunity: increasing plant AsA content not for feeding it to humans, but for the survival and better performance of overproducing plants. This second approach appears more feasible, and generally produced valuable results.

## 3 Ways to increase vitamin C content in plants

Among the different strategies used to increase AsA content in plants with the aim of producing biofortified crops (Strobbe et al., 2018), three main approaches have been used so far: 1. Increasing AsA content by affecting its biosynthesis; 2. Increasing AsA content by improving recycling from its oxidized forms; 3. Increasing AsA content by limiting its catabolism

### 3.1 The long way to the discovery of the AsA biosynthetic pathway in plants

**Figure 1:**
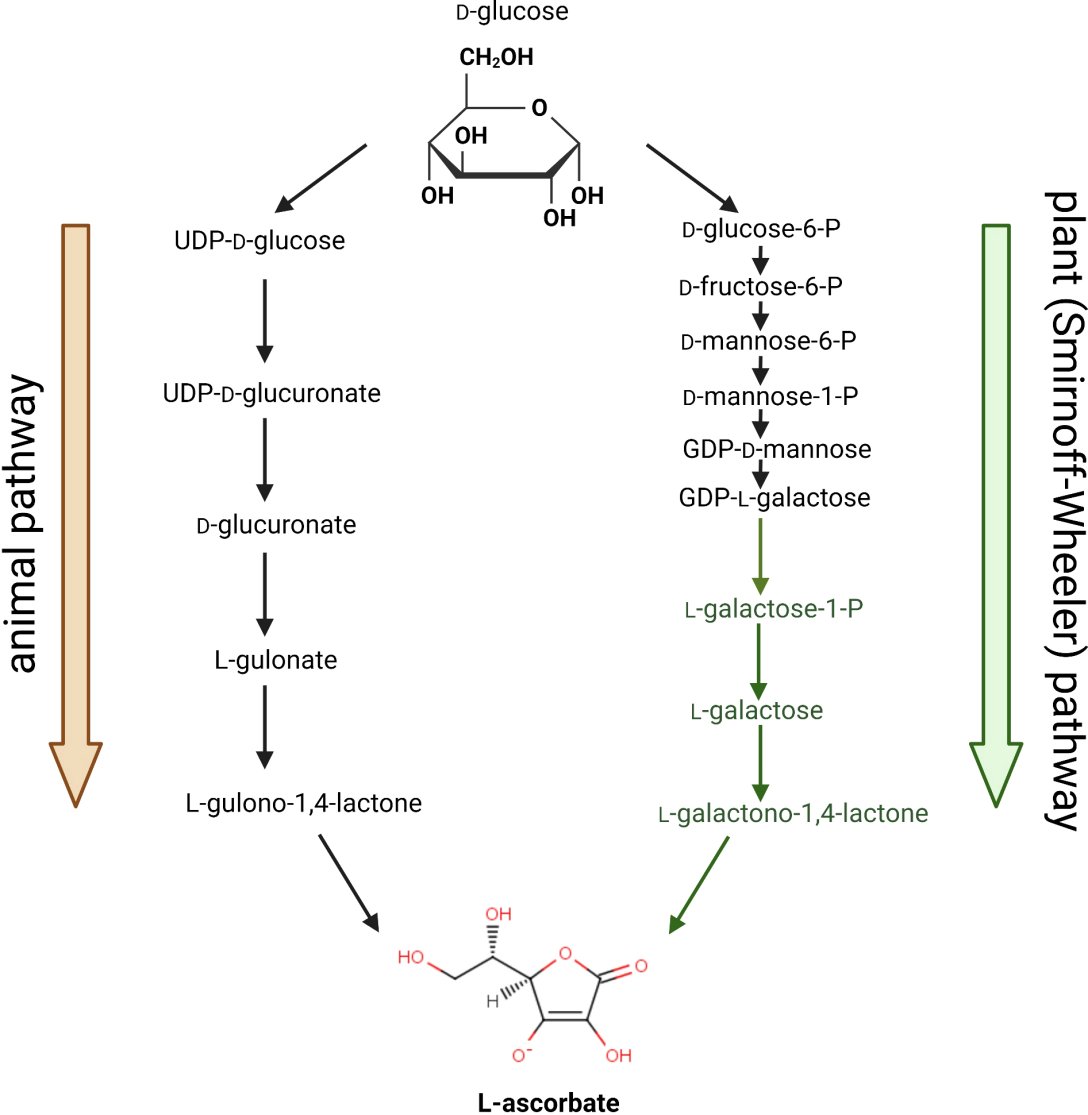
Ascorbic acid (AsA) biosynthetic pathways in animals and in plants. The committed steps (specific for AsA synthesis) in the plant pathway are highlighted in green.

Full elucidation of the AsA biosynthetic pathway in plants has been quite troublesome and took decades of investigation. Early work by Mapson and co-workers in the 1950s (Isherwood et al., 1954) suggested that animals and plants have different biosynthetic routes. The two pathways, as we know them today, are compared in **Figure 1**. For years the debate focused on the inversion *vs.* non-inversion pathway, *i.e.* whether carbon 1 of d-glucose, the initial precursor, becomes carbon 6 in l-AsA (inversion, as in the animal pathway), or is retained as C1 in AsA (non-inversion) (Smirnoff et al., 2001). The final step in the plant pathway, the conversion of l-galactono-γ-lactone (l-GalL) into AsA, was observed in the presence of the mitochondrial fraction (Mapson et al., 1954). The enzyme catalyzing this step was later identified as a mitochondrial dehydrogenase (Ôba et al., 1995), in a difference to the animal pathway, in which the last step is catalyzed by an oxidase associated to the microsomal fraction. Until the turn of the XX Century there was no consensus on all other steps in the pathway, and the situation was quite confused. Still in 1990, the unusual precursors d-glucosone and l-sorbosone were proposed as intermediates in the plant pathway (Saito et al., 1990). Labeling experiments with C-14, which had been so very effective for the study of different pathways, and very helpful even to disentangle the complexity of the Calvin cycle (Bassham et al., 1953), did not work as well in the case of AsA. This is possibly due to the fact that the amount of AsA produced in the pathway is relatively low, and the pathway itself is interconnected to different biosynthetic routes, so the radioactive labeling was scattered onto many different carbohydrate molecules, with no chance to observe clear trends and quantitatively relevant key intermediates. Alternatively, attempts to target enzyme activities in crude extracts or in partially purified protein fractions were also hardly successful: the activities of the enzymes putatively involved in the pathway were apparently quite low, when detectable. Only the outstanding work performed by the Smirnoff group at the University of Exeter in the late 1990s eventually reached the goal of deciphering the full picture of a coherent biosynthetic pathway (Wheeler et al., 1998). Further support to the Smirnoff-Wheeler pathway (also known as the l-galactose pathway) came from genetic evidence obtained by Conklin and Last, who, while looking for ozone-sensitive *Arabidopsis* mutants, observed the *sensitive to ozone1* (*soz1*) mutant, characterized by lower AsA content (about 70-75% less than the wild type). The mutant was soon renamed *vitamin c deficient1* (*vtc1*) when it was found partially defective in the activity of GDP-mannose pyrophosphorylase, the enzyme catalyzing an early step in the biosynthesis (Conklin et al., 1999). Additional *vtc* mutants were identified and characterized Conklin et al., 2000). This was the starting point of a “Renaissance” in AsA research, as Smirnoff, Conklin and the pioneer Loewus entitled their seminal paper summarizing those novel and exciting discoveries (Smirnoff et al., 2001). Since then, all the enzymes catalyzing the different steps of the Smirnoff-Wheeler pathway have been characterized, and their corresponding genes cloned. The list of the enzymes involved includes phosphoglucose isomerase (PGI); phosphomannose isomerase (PMI); phosphomannomutase (PMM); GDP-d-mannose pyrophosphorylase (GMP); GDP-d-mannose 3′,5′ epimerase (GME); GDP-l-galactose phosphorylase (GGP); l-galactose 1-phosphate phosphatase (GPP); l-galactose dehydrogenase (l-GalDH); l-galactono-1,4-lactone dehydrogenase (l-GalLDH).

Possible alternative pathways of AsA biosynthesis have been proposed: the MIOX pathway, using myo-inositol as a precursor (Lorence et al., 2004), and the galacturonic acid pathway (Agius et al., 2003). Rather than full pathways, they both are alternate entry points into the biosynthetic machinery and might contribute to building the AsA pool under some circumstances, but they are unlikely to provide the bulk amount of AsA required by the cell. An additional pathway going from GDP-d-mannose to l-gulonic acid, through GDP-l-gulose, l-gulose-1-P and l-gulose, has also been proposed (Valpuesta and Botella, 2004).

### 3.2 Increasing AsA content by modulating its biosynthesis is not easy

Over the years, virtually all the genes involved in the Smirnoff-Wheeler pathway have been overexpressed under the control of constitutive promoters to obtain higher AsA content in different plant species, but in almost all cases the actual results were far below the expected (Macknight et al., 2017; Strobbe et al., 2018), the only valuable exception being *GGP* (**Table 2**). Pyramiding tomato lines in which 4 genes in the pathway (*GME*, *GMP*, *GGP*, and *GPP*) were co-overexpressed under the control of the 35S CaMV promoter showed increased AsA content in leaves and, to lesser extent, in fruits, but co-overexpression was only slightly more effective than other gene combinations (Li et al., 2019). When data on AsA increase in the overexpressors are reported as fold change compared to controls (untransformed or mock-transformed plants), the results may appear promising (see *e.g.* Macknight et al., 2017), but if we go into details and check the actual AsA content measured (**Table 2**) and the variability within the same population of transformants, we realize how far we are from the goal insistently claimed in almost all the papers reporting such experiments: meeting the needs of human nutrition.

**Table 2:** Effect of the overexpression of selected genes of the main ascorbic acid (AsA) biosynthetic pathway on AsA content.

Gene(s) overexpressed	Plant species and organ	AsA content in control plants	AsA content and fold increase in transformants	Ref.
*GMP = GDP-d-mannose pyrophosphorylase (VTC1)*	*Arabidopsis thaliana* leaf	0.358 ± 0.086 mg/g fw	0.508 ± 0.084 mg/g fw (1.4)	Zhou et al., 2012
*GME = GDP-d-mannose 3′,5′ epimerase*	*A. thaliana* leaf	0.358 ± 0.086 mg/g fw	0.497 ± 0.095 mg/g fw (1.3)	Zhou et al., 2012
*GGP = GDP-l-galactose phosphorylase (VTC2)*	Potato tuber Tomato fruit Strawberry fruit	0.50 ± 0.2 mg/g dw 0.18 ± 0.4 mg/g fw 0.62 ± 0.1 mg/g fw	1.65 ± 0.47 mg/g dw (3) 1.11 ± 0.23 mg/g fw (6) 1.31 ± 0.1 mg/g fw (2)	Bulley et al., 2012
*GPP = l-galactose 1-phosphate phosphatase (VTC4)*	*A. thaliana* leaf	0.358 ± 0.086 mg/g fw	0.572 ± 0.102 mg/g fw (1.6)	Zhou et al., 2012
*l-GalDH = l-galactose dehydrogenase*	*A. thaliana* leaf	0.358 ± 0.086 mg/g fw	0.468 ± 0.081 mg/g fw (1.3)	Zhou et al., 2012
*l-GalLDH = l -galactono-1,4-lactone dehydrogenase*	*A. thaliana* leaf	0.358 ± 0.086 mg/g fw	0.647 ± 0.143 mg/g fw (1.6)	Zhou et al., 2012
*GMP+GME+GGP+* *GPP*	Tomato leaf Tomato fruit	~ 0.4 mg/g fw ~ 0.28 mg/g fw	~ 1 mg/g fw (2.5) ~ 0.37 mg/g fw (1.3)	Li et al., 2019

For some genes, the names of the corresponding *Vitamin C deficient* (*VTC*) genes of *Arabidopsis* are reported. fw: fresh weight; dw: dry weight.

Manipulation of AsA content by tackling alternate pathways was not more effective. Overexpression of the strawberry d-galacturonic acid reductase gene in tomato increased fruit AsA content from about 2 to about 5 mg/100g f.w. (Lim et al., 2016), meaning that an adult should eat almost 2 kilograms of tomatoes per day to fulfill the prescribed AsA requirement (**Table 1**). It is surprising that so many attempts have been made to increase AsA content in tomato plants, considering that organs of different species (fruits of *Capsicum annuum*, various *Citrus* species, *Fragaria* sp. and inflorescences of *Brassica oleracea*) make better sources of AsA (Palma et al., 2015).

Expression of an animal (from rat liver) cDNA encoding l-gulonolactone oxidase increased AsA content in lettuce (Jain and Nessler, 2000), but the use of non-plant genes to boost AsA contents in plants had little further development, conceivably because similar products would hardly be accepted by consumers and have any market.

### 3.3 A closer look into GGP

As mentioned above, several reports confirm that GDP-l-galactose phosphorylase (GGP) catalyzes a key step in AsA biosynthesis. The identification of this enzyme as the product of the *Arabidopsis* gene *VTC2* was reported independently by three research groups in the same year (Dowdle et al., 2007; Laing et al., 2007; Linster et al., 2007). In the Smirnoff-Wheeler pathway, GGP is the first committed step in AsA production, as the previous steps provide GDP-d-mannose and l-galactose, that are also used in cell wall metabolism (Smirnoff et al., 2001). Dowdle et al. (2007) also found an *Arabidopsis* gene (*VTC5*) coding for a second GGP with distinct kinetic features, as compared to the VTC2 enzyme. Interestingly, seeds of the double mutant *vtc1/vtc5* can germinate, but seedlings undergo early growth arrest and die if not supplemented with exogenous AsA, demonstrating that “scurvy” plants are not viable. Most likely, the germination of the *vtc2/vtc5* double mutant is made possible by the reduction of dehydroascorbic acid to AsA by means of dehydroascorbate (DHA) reductase (see below), since it has been demonstrated and repeatedly confirmed that mature orthodox seeds have no AsA (reduced form) and retain only a small amount of DHA (oxidized form) (Arrigoni et al., 1992).

Overexpression of *GGP* mostly (but not always) increased AsA content in model and non-model plant species (Macknight et al., 2017; Broad et al., 2020). Soon after its characterization in 2007, it was very clear that GGP has a special regulatory role in the AsA biosynthetic pathway and is a strategic target for further research. An interesting study on *VTC2* in *Arabidopsis* (Müller-Moulé, 2008) showed that the expression of the gene is rapidly elicited by light in green tissues, whereas root expression is much lower. Most interestingly, and somewhat surprisingly, the YFP-tagged VTC2 protein localizes not only to the cytosol, but also to the nucleus. Nuclear, in addition to cytoplasmic location, has been observed also for some other enzymes of the Smirnoff-Wheeler pathway, namely GMP, GPP, and l-GalDH (Fenech et al., 2021). This finding suggests a regulatory role for such proteins, but further studies will be necessary to elucidate this point. An accurate and comprehensive kinetic model of AsA biosynthesis confirmed that GGP is the main control point and limiting step of the metabolic flux along the pathway (Fenech et al., 2021).

### 3.4 Regulators of the pathway

Both overexpression studies and data on the effects of feeding with precursors (Pallanca and Smirnoff, 2000; Bulley et al., 2021) confirm that the AsA biosynthetic machinery is tightly regulated. This finding is not consistent with the common opinion that unlimited antioxidant (and in particular AsA) supply is always beneficial to all organisms. AsA inhibits its *de novo* biosynthesis with a typical negative feedback mechanism, apparently affecting three enzymes in the pathway: PMI, GGP, and l-GalDH (Fenech et al., 2021). The search for factors regulating AsA biosynthesis started with the identification by the Nessler group of an *Arabidopsis* phosphatase possibly activating the myo-inositol alternative branch (Zhang et al., 2008). Later on, the *Arabidopsis* VTC3 protein was identified by Conklin et al. (2013) as a putative plastid-associated factor with a dual protein kinase::protein phosphatase function, apparently involved in the light-mediated induction of AsA biosynthesis: notably, the *vtc3* mutant shows no increase in AsA content in response to light and heat (Conklin et al., 2013). The list of transcription factors putatively involved in AsA synthesis includes the ethylene response factor AtERF98 (Zhang et al., 2012), the tomato SlHZ24 (Hu et al., 2016) and SlDOF22 (Cai et al., 2016). Two more regulatory factors apparently activate the VTC1 protein (the *Arabidopsis* GMP): KONJAC (Sawake et al., 2015) and CSN5B (Wang et al., 2013), the latter being involved in the ubiquitination and subsequent degradation of VTC1. An F-box protein also controls *GMP* expression in *Malus domestica* (Ma SY et al., 2022). An *Arabidopsis* calmodulin (CML10) has been found to interact with *PMM*, with a possible regulatory role (Cho et al., 2016).

A real breakthrough in the search for factors controlling AsA biosynthesis occurred when Laing et al. (2015) reported on a *cis*-acting upstream open reading frame (uORF) repressing the translation of the downstream *GGP* open reading frame under high ascorbate concentration. The peptide encoded by the noncanonical uORF functions in the ascorbate-induced inhibition of translation. Disruption of the uORF using a CRISPR-Cas9 approach removed the AsA feedback repression of *GGP* and increased AsA content in tomato fruit up to the remarkable amount of 1 mg per gram fresh weight (Deslous et al., 2021). Two more transcription factors from *Actinidia eriantha* (AceMYBS1 and AceGBF3) working synergistically to activate *GGP* have been recently described by Liu et al. (2022). Interestingly, AceMYBS1 is repressed by abscisic acid (ABA), confirming that AsA biosynthesis is also hormone-regulated. Wolucka et al. (2005) observed stimulation of AsA biosynthesis in methyl-jasmonate-treated tobacco and *Arabidopsis* suspension cultures. Additionally, the signaling molecule nitric oxide (NO) caused a 40% AsA increase in pepper fruit, possibly by influencing l-GalLDH activity (Rodríguez-Ruiz et al., 2017; Zuccarelli et al., 2021). Increasing AsA content by using treatments with growth regulators could be a useful alternative to invasive gene manipulation techniques.

### 3.5 The Smirnoff-Wheeler pathway connects different cell components and signaling modules

**Figure 2:**
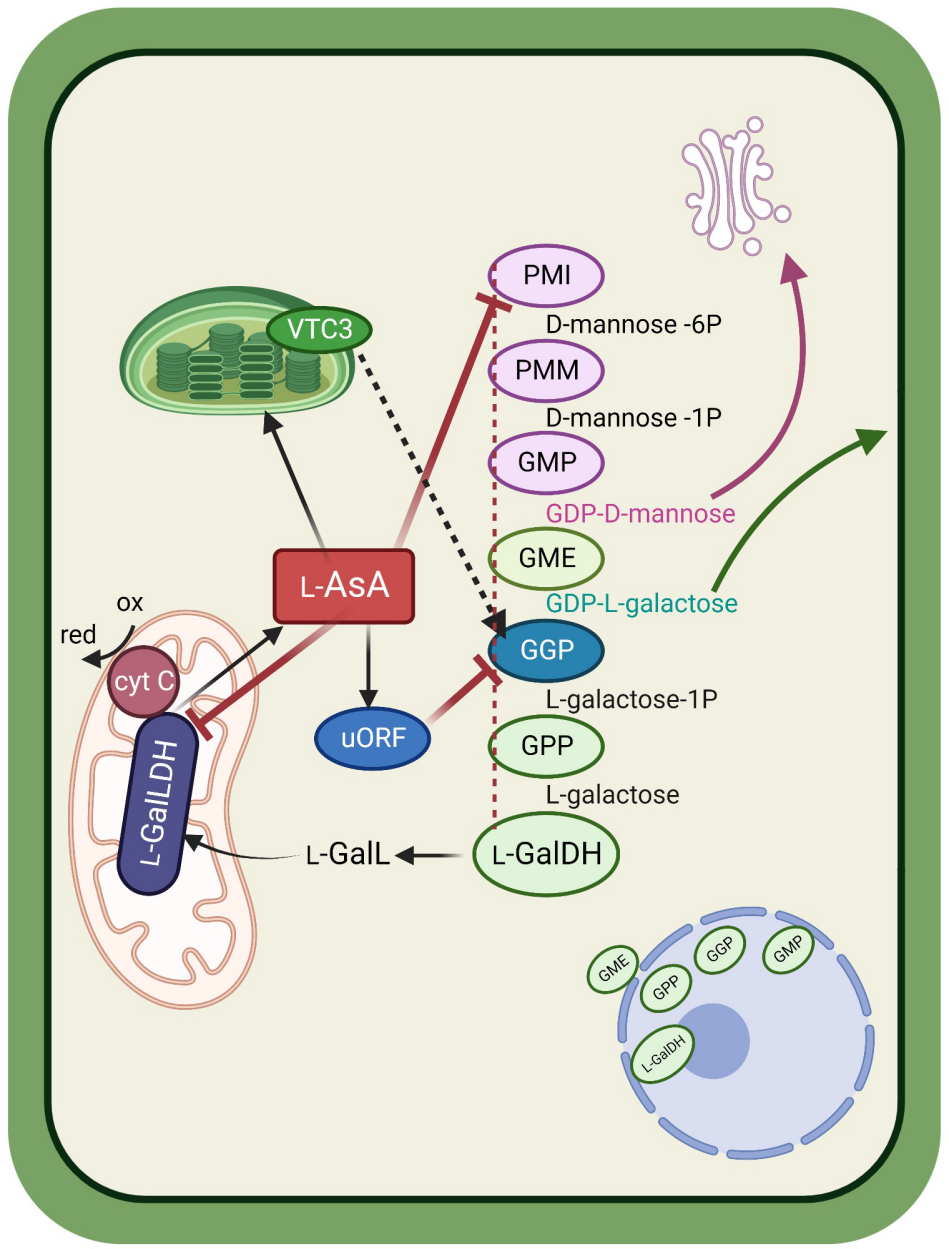
Schematic representation showing the main ascorbic acid (AsA) biosynthetic pathway in its cellular context. Cytosolic enzymes in the Smirnoff-Wheeler pathway, possibly physically connected to form a multienzyme complex (red dotted line) produce intermediates used for glycoprotein (in the Golgi) and cell wall assembly. The committed steps in the pathway yield l-galactono-1,4-lactone that is transported to the mitochondrion, where a specific dehydrogenase catalyzes its conversion to AsA using oxidized cytochrome c as an electron acceptor. AsA controls its biosynthesis through feedback inhibition on three enzymes of the pathway. Inhibition of GGP activity is mediated by an AsA-regulated upstream Open Reading Frame (uORF). The chloroplast-associated factor VTC3 could be associated to light-dependent activation of GGP (black dotted line). Nuclear co-location of 4 enzymes of the pathway could have a yet unexplored regulatory role. PMI=phosphomannose isomerase; PMM=phosphomannomutase; GMP=GDP-d-mannose pyrophosphorylase; GME=GDP-d-mannose 3′,5′ epimerase; GGP=GDP-l-galactose phosphorylase; GPP= l -galactose 1-phosphate phosphatase; l-GalDH= l-galactose dehydrogenase; l-GalLDH= l-galactono-1,4-lactone dehydrogenase.

The last ten years have brought quite a lot of relevant information on the regulation of the Smirnoff-Wheeler pathway. Although there is still much to understand about how the pathway actually works, the picture is getting clearer and clearer. A tentative model is described in **Figure 2**. The first steps in the pathway serve two different purposes: producing intermediates that will go through the pathway to yield AsA, and producing key molecules that will eventually be targeted to the cell wall, namely d-mannose and l-galactose. The reaction catalyzed by GGP is the first committed step, and the 3 final ones, catalyzed by GPP, l-GalDH and l-GalLDH, respectively, are specific for AsA production. All the reactions are known to occur in the cytosol, with one major exception: the peculiar location of the last enzyme, l-GalLDH, at the mitochondrial inner membrane (Siendones et al., 1999). If we add to the picture the involvement in AsA synthesis of the plastid-located factor VTC3 (Conklin et al., 2013), and the observed co-presence in the nucleus of the cytosolic enzymes GMP, GGP, GPP, and l-GalDH (Müller-Moulé, 2008; Fenech et al., 2021), we can conclude that AsA biosynthesis spans all over the cell establishing connections between cell wall metabolism, photosynthesis and respiration, all processes exerting an obvious regulatory influence on AsA production. There is evidence that all the enzymes in the pathway, of course with the exception of the mitochondrial one, could be connected and form a single multienzyme complex (Fenech et al., 2021). This possibility (represented by the red dotted line in **Figure 2**) deserves further investigation.

Plants deficient in GMP activity show interesting features. Reduced growth and early senescence were observed in antisense potato plants underexpressing a *GMP* gene, in parallel with lower AsA content (-60%) and cell wall-associated mannose (-70%) in leaves, while tubers develop normally (Keller et al., 1999). Decreased growth and delayed flowering also occur in the *Arabidopsis vtc1* mutant, defective in GMP activity (Veljovic-Jovanovic et al., 2001). However, the growth defects of these plants are likely due to the reduced availability not only of AsA, but also of mannose. The relevance of GMP activity is also indirectly witnessed by the presence of specific regulators interacting with the enzyme (Sawake et al., 2015). Partial inactivation of GMP activity in the *Arabidopsis hsn1* mutant, allelic to *vtc1*, caused ammonium hypersensitivity not due to the partial loss of AsA, but entirely to impaired N-glycosylation. Moreover, ${\text{NH}}_{4}^{+}$ inhibits GMP activity (Qin et al., 2008).

The final step of the biosynthesis takes place in the mitochondrion. The reaction catalyzed by l-GalLDH uses cytochrome c as an electron acceptor (Ôba et al., 1995; Leferink et al., 2008). From a structural point of view, the protein regulates the assembly of the mitochondrial complex I (Schimmeyer et al., 2016). The correlation between respiratory electron transport and AsA synthesis has been widely investigated (Millar et al., 2003; Szarka et al., 2013). Imbalance of AsA biosynthesis also affects photosynthetic activity (Senn et al., 2016). It is especially intriguing that silencing of l-GalLDH had no effect on total AsA+DHA content, but reduced leaf and fruit size in tomato plants, also affecting the TCA cycle and secondary metabolic pathways related to stress response (Alhagdow et al., 2007).

### 3.6 Additional ways to increase AsA content: MDHA/DHA reductases and AsA oxidase

In the classical Halliwell-Asada pathway, AsA oxidized forms (namely monodehydroascorbate, MDHA, and dehydroascorbate, DHA) can be reduced back to AsA by means of the enzymes MDHA reductase (MDHAR) and DHA reductase (DHAR, respectively (Hausladen and Kunert, 1990). The possibility of increasing AsA content by overexpressing the two enzymes has been explored over the years. MDHA is a short-lived free radical (also known as Ascorbate Free Radical) that is considered to disproportionate (in an uncatalyzed reaction) yielding AsA and DHA, whereas the enzyme MDHAR is NADH-dependent. In turn, DHA is reduced back to AsA in a glutathione-dependent reaction catalyzed by DHAR (Arrigoni et al., 1981). A MDHAR-coding gene from *Malpighia glabra* (acerola) has been expressed in tobacco leaves with a 1.8-fold increase in AsA content, but once more, the basal AsA level is relatively low, in the nanomoles per gram fresh weight range (Eltelib et al., 2012). Often erroneously represented as a tricarbonyl molecule (Kerber, 2008), DHA in aqueous solution is actually a dimer, whereas the tricarbonyl form, named pseudodehydroascorbic acid, is very unlikely to occur because of its extreme instability (Njus et al., 2020). The presence of a specific enzyme catalyzing the reduction of DHA has been a matter of debate, because several proteins sharing a C-X-X-C motif can potentially act as DHARs (Morell et al., 1997; Morell et al., 1998). Later on, putative DHARs have been cloned and characterized (Urano et al., 2000), and in some cases overexpressed with the aim of increasing AsA content (Lin et al., 2016). Also in the case of DHARs, the results of the overexpression on AsA content were far from dramatic. It should be considered that, during plant development, DHAR activity is usually not consistent with AsA content (De Tullio et al., 1998; Lin et al., 2016). Most likely, DHARs act as modulators of DHA content rather than significantly contributing to the AsA pool, with the exception, already mentioned above, of seed germination.

In principle, the AsA pool can be increased also by limiting its degradation. A possible strategy to increase AsA content might be the targeting AsA oxidase (AO), an enzyme of still unclear physiological role (De Tullio et al., 2013). The suppression of AO expression in antisense tobacco plants and in a T-DNA insertion *Arabidopsis* mutant (Yamamoto et al., 2005) resulted in higher AsA content (approximately up to 3-fold increase). However, virus-induced AO silencing in tomato plants increased fruit yield under water limiting conditions, but hardly affected AsA content (Garchery et al., 2013).

## 4 The consequences of increasing AsA content: All that glitters is not gold

All the attempts made to increase AsA content in plants are based on the assumption that more AsA can only be beneficial to plants and, by consequence, to the humans who are supposed to consume those “biofortified” plants in their diet. No matter how much AsA is produced, no negative outcome will ever occur. This principle, possibly a consequence of the popularization of Pauling’s hypotheses unproperly transferred to plants, is contradicted by the simple observation that AsA biosynthesis is strictly regulated. As discussed above, the biosynthetic pathway leading to AsA production is very hard to “crack”, and only in very few cases, over a large number of attempts, AsA content in tomato fruit was raised up to a level that could, at least in theory, be used for the needs of human nutrition (Bulley et al., 2012; Deslous et al., 2021). From an evolutionary point of view, it is unconceivable that AsA synthesis is kept on a tight leash without any selective advantage. Is there an unexpected trade-off behind the limitations in plant AsA production? Wheeler et al. (2015) in their comprehensive, excellent study on the evolution of AsA biosynthesis, showed that in eukaryotes the ancestral gene gulono-lactone oxidase (*GULO*), encoding the enzyme still present in the animal biosynthetic pathway, was lost in early photoautotrophs and replaced by l *-GalLDH* in the *Archaeplastida* lineage, but also in some non-photosynthetic organisms that have lost their plastids. The plant enzyme apparently confers an advantage over the animal one, also in the view that photosynthesis unavoidably produces an excess of reactive oxygen species that need effective management (Maruta et al., 2016). The presence in plants of AsA peroxidases, ubiquitary in all cell compartments, suggests that plants require more AsA than animals, but the question whether an “upper limit” to AsA biosynthesis might occur in plants still stands.

A closer look at tomato plants produced by *GGP* overexpression (Bulley et al., 2012), or removal of the uORF controlling *GGP* expression (Deslous et al., 2021) shows surprising and interesting anomalies in fruit development. In tomato lines with high AsA content due to the expression of the kiwifruit *GGP* gene under the control of the 35S promoter, the fruits were not only smaller and lighter (16 ± 2 g in the line with 6-fold AsA increase, as compared to 52 ± 8 g in control plants), but they were also seedless (or had small, nonviable seeds), and the typical mucilage in locular tissue was missing (Bulley et al., 2012). Similarly, the removal of the AsA-regulated feedback inhibition either by mutagenesis or by using a CRISPR/Cas9 approach (Deslous et al., 2021) markedly increased AsA content in tomato plants, but concomitantly caused the production of seedless fruits (and in some cases no fruit production at all) in homozygous lines. The seedless phenotype cannot be ascribed to a switch to parthenocarpy, but is clearly due to male sterility, since further analysis of the AsA-overproducing plants showed altered anther development and anomalous pollen grains unable to produce a functional pollen tube. The phenotype of heterozygous plants was somewhat intermediate. Transcriptome analysis evidenced effects of AsA overproduction mainly on defense responses and the immune system, suggesting a trade-off between defense and development (Deslous et al., 2021).

Previous studies, in which AsA content had been increased by feeding *Arabidopsis* plants with AsA precursors, (mainly l-GalL), have shown that AsA affects plant development by delaying the transition to the reproductive stage (Attolico and De Tullio, 2006; Barth et al., 2006). The precursor-feeding approach has been recently used to investigate the effect of increased AsA content on the expression of possible downstream targets and regulators (Bulley et al., 2021), evidencing a putative regulatory network that mainly involves ABA responsive genes. ABA content and the expression of the gene encoding the ABA biosynthetic enzyme NCED3 are also increased by AsA enhancement. Notably, NCEDs (9’-*cis*-epoxycarotenoid dioxygenases) are enzymes belonging to the large class of dioxygenases. Many dioxygenases share a complex catalytic mechanism in which AsA has a key regulatory role (De Tullio, 2020).

## 5 Conclusions: A long way ahead

In the last 10-15 years, astonishing progress has been made in the development of powerful tools allowing us to manipulate genes and genomes and introduce favorable traits in plants. Such improved techniques, and mainly the apparently unlimited potential of the CRISPR/Cas9 approach, are now opening new stimulating perspectives for the production of healthy crops able to meet the needs of a growing world population. However, to avoid wasting time and money, careful planning should be made before starting a research project aimed at altering a key component in plant metabolism. At least at the moment, increasing vitamin C content to obtain superfoods and defeat any disease just by eating good stuff sounds like pure hype, for two different reasons. First, the popular claim that AsA is always good, no matter how much, should probably be re-considered (Tóth et al., 2018). Moreover, as discussed above, the “perfect crop” able to provide unlimited vitamin C supply at low cost is very unlikely to be obtained in the near future.

Even more than other known biosynthetic routes, AsA biosynthesis appears entangled in the complexity of cell metabolism. Unbalancing AsA content beyond a certain threshold resulted in anomalies in reproductive development. It is especially interesting that Deslous et al. (2021) boosted AsA content beyond the “safe” limit not by heterologous overexpression of *GGP* (as in Bulley et al., 2012), but by removing the block that keeps *GGP* under the AsA regulated feedback control, thus “unleashing” the Smirnoff-Wheeler pathway. This means that the “leash” is not there by accident, and that AsA must be handled with care in different cell locations and developmental stages. A clear example is the absence of AsA from dry orthodox seeds (Arrigoni et al., 1992). Incidentally, this is a major challenge if one wishes to make vitamin C available to the entire world population, since dry seeds of some species are staple foods of uttermost importance (Strobbe et al., 2018). It is tempting to speculate that low vitamin C availability is necessary whenever cell metabolism must be downregulated, as in seeds or in the quiescent center of the root apical meristem, where AsA is apparently “leashed” by AsA oxidase (Kerk and Feldman, 1995; Liso et al., 2004; De Tullio et al., 2010). A specific involvement of AsA in the epigenetic control of gene expression *via* the demethylation of DNA and histone proteins catalyzed by TET dioxygenases has been reported (Young et al., 2015). This regulatory mechanism, initially observed in animals, is now known to be widespread in plants too (Ma S et al., 2022). Altering AsA content is likely to affect the epigenome, which conceivably results in changes in the plant developmental program.

When describing the phenotype of GPP-overexpressing plants, Bulley et al. (2012) underline that seedless fruits occur in tomato, but not in strawberry plants transformed with the same constructs. Nonetheless, in the strawberry line with the highest GPP activity, fruit size is apparently lower (unfortunately no picture of the strawberries is shown in the paper). Indeed, in the mentioned paper strawberries have higher basal AsA than tomato fruits also in the control plants, and the fold change in AsA content observed after the transformation is definitely lower (2-fold change in strawberries, 6-fold in tomatoes). This observation raises the question whether different species could have a different “threshold value” beyond which AsA becomes too much. Although at the moment there is no answer, the simple observation that AsA is unevenly distributed among different organs, tissues, cells and even cell compartments (Zechmann, 2018) suggests that AsA is produced “on demand” and transported to the sites of utilization, where it has been suggested to operate as a redox buffer (Palma et al., 2015). The light-dependency of AsA biosynthesis is a clear example: green tissues require AsA upon activation of the photosynthetic process, and AsA is delivered to the chloroplast to scavenge excess reactive oxygen species. VTC3, which is plastid located, is possibly involved in the signaling module activating AsA biosynthesis at the *GGP* level (**Figure 2**). The reason why some plant species have high vitamin C content in the fruit, mostly fleshy fruits used for animal consumption, is another intriguing issue. Recent studies have demonstrated that the nutrient content in fleshy fruits, together with other fruit features, can influence the choices of frugivore bats and birds (Rojas et al., 2021a; Rojas et al., 2021b). Notably, some bats and birds, similarly to humans and some other primates, have lost their AsA biosynthetic capability. The possibility that high AsA in the fruit evolved as a form of reward for the animals involved in seed dispersal, as nectar is a reward for pollinators, deserves further investigation, although at the moment this is just a hypothesis.

Although, as discussed above, almost all attempts to enhance AsA production by modulating the expression of AsA biosynthetic genes did not result in dramatic changes in AsA content, even moderate increases appeared beneficial for plant resistance to different forms of abiotic stress. This is an interesting and useful notion *per se*, but quite often it is explained exclusively as the result of the antioxidant action of AsA, without further investigation. Undoubtedly a strong connection exists between AsA and defense responses, as evidenced by studies on the transcriptome of plants with altered AsA content (Pastori et al., 2003; Deslous et al., 2021). To this respect, the identification of a possible ABA-dependent signaling module regulating AsA biosynthesis (Liu et al., 2022) is especially interesting, as it goes beyond the usual unfocused concept of a fight between “good” antioxidants and “bad” ROS. The hypothesis that AsA is a key player in the trade-off between defense and development, as suggested by Deslous et al. (2021) is probably the best direction to pursue in the next years of AsA research.

## Author contributions

MT performed a wide literature search, edited the manuscript, and cooperated in the preparation of tables and figures. MD conceived the article and the figures, and wrote the manuscript. All authors contributed to the article and approved the submitted version.
